# Efficacy of Humanized Cefiderocol Exposures over 72 Hours against a Diverse Group of Gram-Negative Isolates in the Neutropenic Murine Thigh Infection Model

**DOI:** 10.1128/AAC.01040-18

**Published:** 2019-01-29

**Authors:** Sean M. Stainton, Marguerite L. Monogue, Masakatsu Tsuji, Yoshinori Yamano, Roger Echols, David P. Nicolau

**Affiliations:** a Center for Anti-Infective Research and Development, Hartford Hospital, Hartford, Connecticut, USA; b Lake Erie College of Osteopathic Medicine, School of Pharmacy, Erie, Pennsylvania, USA; c Shionogi & Co., Ltd., Osaka, Japan; d ID3C, LLC, Easton, Connecticut, USA; e Division of Infectious Diseases, Hartford Hospital, Hartford, Connecticut, USA

**Keywords:** Gram-negative bacteria, pharmacodynamics, pharmacokinetics

## Abstract

Herein, we evaluated sustainability of humanized exposures of cefiderocol *in vivo* over 72 h against pathogens with cefiderocol MICs of 0.5 to 16 μg/ml in the neutropenic murine thigh model. In Acinetobacter baumannii, Pseudomonas aeruginosa, and Enterobacteriaceae displaying MICs of 0.5 to 8 μg/ml (*n* = 11), sustained kill was observed at 72 h among 9 isolates. Postexposure MICs revealed a single 2-dilution increase in one animal compared with controls (1/54 samples, 1.8%) at 72 h. Adaptive resistance during therapy was not observed.

## Abstract (precis)

Herein, we evaluated sustainability of humanized exposures of cefiderocol *in vivo* over 72 h against pathogens with cefiderocol MICs of 0.5 to 16 μg/ml in the neutropenic murine thigh model. In Acinetobacter baumannii, Pseudomonas aeruginosa, and Enterobacteriaceae displaying MICs of 0.5 to 8 μg/ml (*n* = 11), sustained kill was observed at 72 h among 9 isolates.

## INTRODUCTION

Standard pharmacodynamic assessment studies designed to quantify antimicrobial efficacy typically occur over 24 h. However, prolonged (i.e., 72-h) studies have demonstrated additional insight into the characterization of a drug’s rate, extent, and robustness of antimicrobial activity (1, 2). The effect of humanized doses of cefiderocol, a novel siderophore cephalosporin, was recently characterized across a wide array of clinical isolates in the neutropenic murine thigh infection model. Data from these 24-h pharmacodynamic studies revealed that cefiderocol humanized exposures produced predictable bacterial kill against multidrug resistant (MDR) Gram-negative isolates with MICs (MICs) of ≤4 μg/ml (3). Little is known about the sustained antimicrobial effect of cefiderocol beyond 24 h. Furthermore, previous studies with siderophore monobactams demonstrated adaptive resistance in the presence of antibiotic pressure (4, 5). The aim of the current study was to evaluate the sustainability of cefiderocol activity *in vivo* over 72 h against Gram-negative pathogens and to characterize the phenotypic and genotypic profiles of organisms recovered postexposure.

Shionogi & Co., Ltd., supplied cefiderocol vials (lot 12M01) for the purposes of *in vivo* testing. The cefiderocol dosing regimen utilized has been validated in several studies with the same mouse model to provide exposures equivalent to those in humans attained after administration of 2 g every 8 h (q8h) (3 h infusion) (3, 6, 7). Commercially available cefepime acquired from Cardinal Health, Inc. (1 g; lot 106014C; Sagent Pharmaceuticals) was also utilized. Cefepime vials were reconstituted and diluted with 0.9% normal saline and administered in volumes of 0.2 ml as subcutaneous injections at doses simulating those equivalent to 2 g q8h (3 h infusion) in humans (7).

A total of 12 Gram-negative isolates (2 Pseudomonas aeruginosa isolates, 4 Acinetobacter baumannii isolates, and 6 Enterobacteriaceae isolates) with cefiderocol MICs ranging from 0.5 to 16 μg/ml were provided by Shionogi & Co., Ltd., or IHMA, Inc. These isolates were selected from among those utilized in the previous 24-h pharmacodynamic study representing various species across a range of cefiderocol MICs, having shown modest efficacy despite lower MICs to cefiderocol (3). By use of iron-depleted cation-adjusted Mueller-Hinton broth (ID-CAMHB), as recommended by CLSI, isolates were screened for cefiderocol MICs in triplicate, and the modal value was reported (8). Cefepime MICs were determined in triplicate according to CLSI broth microdilution methods (9). For isolates shown to regrow at 72 h or to otherwise display an unexpected response (ranging from stasis to 4 log growth) despite cefiderocol MICs predictive of susceptibility (i.e., KP 543, EC 458, KP 539, AB 152, EC 462, KP 531), a bacterial sample from a single thigh of treated and control animals was collected and tested for its cefiderocol postexposure MIC at 24, 48, and 72 h. Any change in MIC of >2-fold compared with those in infected controls in the same period of exposure was considered meaningful. All retest MICs were performed in triplicate using ID-CAMHB.

Subsequent whole-genome sequencing was performed on bacterial isolates after cefiderocol exposure and on paired *in vivo* controls. All genotypic analyses were performed with the CLC genomics workbench (version 11; Qiagen). Reads from whole-genome shotgun sequencing on an Illumina HiSeq (150-bp paired-end reads) were sampled down to a predicted coverage depth of 75× for each genome, and *de novo* assembly was performed. β-Lactamase genes were identified by use of the “find resistance” module, which utilizes the same database as that utilized by ResFinder (https://cge.cbs.dtu.dk/services/ResFinder/). All β-lactamase genes identified had 100% nucleotide sequence identity and length of the specified reference except for the endogenous class C β-lactamase gene of Escherichia coli, *bla*_EC_, which had 97% identity to the reference in each isolate. MLST was carried out on a guided assembly utilizing the most closely related closed genome in NCBI (Find Best Matches using K-mer Spectra tool) as the reference sequence. A minimum coverage depth of 30× for each of the 7 loci was obtained. MLST databases were acquired from PubMLST (https://pubmlst.org/), and the Achtman scheme was used for E. coli isolates. Genome assemblies from the two control isolates, 32a (E. coli) and 73a (Klebsiella pneumoniae), were annotated with the rapid annotation using subsystem technology (RAST) pipeline (10–12).

Reads from the treated-isolate genomes were aligned to the annotated contigs of their respective control isolates, and the Basic Variant Detection tool was used to identify single nucleotide polymorphisms (SNPs) and small in/del mutations in the treated isolates. Positions with <20× or >300× read depths were ignored. If the mutation had an impact on the sequence of an annotated gene, the resultant amino acid variation was indicated. Reads from samples 32a and 73a were aligned to their own annotated assemblies for mutational analysis as described above. Any mutations identified this way were considered spurious and were not reported when identified in the genomes of the treated strains.

The study protocol was reviewed and approved by the Institutional Animal Care and Use Committee at Hartford Hospital. Animals were prepared by using a previously described murine thigh infection model (13).

All isolates were stored frozen at −80°C in skim milk (BD BioSciences, Sparks, MD). Before mouse thigh inoculation, two bacterial transfers were performed onto Trypticase soy agar plates containing 5% sheep blood (TSA II; Becton, Dickinson & Co., Sparks, MD). Subsequently, a suspension containing ∼10^7^ CFU/ml was made for inoculation. Thigh infection was established via intramuscular injection of the inoculum (0.1 ml) into each thigh 2 h before the initiation of antimicrobial therapy.

In total, 12 isolates (2 P. aeruginosa [MIC range, 2 to 4 μg/ml], 4 A. baumannii [MIC range, 0.5 to 16 μg/ml], and 6 Enterobacteriaceae [MIC range, 1 to 8 μg/ml]) were tested in the neutropenic thigh infection model. Cefiderocol preexposure and comparator MIC values are presented in Table 1. Control animals were dosed with vehicle. For isolates treated only with cefiderocol (i.e., no cefepime control), groups of 3 animals were assigned to 0-h control and to control, cefiderocol, or cefepime groups corresponding to 24-, 48-, and 72-h time points. Animals that did not survive to the time allotted were grouped with the interval corresponding to the time at which they were found deceased. After the animals were killed, the thighs were removed and individually homogenized in normal saline. Samples were serially diluted and plated on agar medium for enumeration of bacterial burden. Changes in CFU for each thigh relative to 0 h were determined at 24, 48, and 72 h, and average values (± standard deviations) were determined for each group. Efficacy was defined as a reduction in bacterial burden relative to 0 h control.

**TABLE 1:** MICs of preexposure cefiderocol and comparators against a collection of 12 *P. aeruginosa*, *A. baumannii*, and *Enterobacteriaceae* isolates

Isolate	MIC (μg/ml) for^*a*^:				
	Cefiderocol	FEP	MEM	CAZ	TZP
AB 135	0.5	ND	128	ND	ND
AB 152	1	64	128	ND	ND
EC 462	1	>64	0.06	>128	>128
EC 458	1	512	0.06	>128	>64
PSA 1574	2	8	16	ND	ND
PSA 1595	4	2	1	ND	ND
KP 531	4	>64	256	>64	>32
KP 519	4	>64	512	>32	>32
KP 539	4	>512	0.12	128	128
AB 87	4	>16	256	>16	>64
KP 543	8	>512	ND	ND	ND
AB 84	16	>64	64	>16	>64

a MEM, meropenem; FEP, cefepime; CAZ, ceftazidime; TZP, piperacillin-tazobactam; ND, no data.

**FIG 1:**
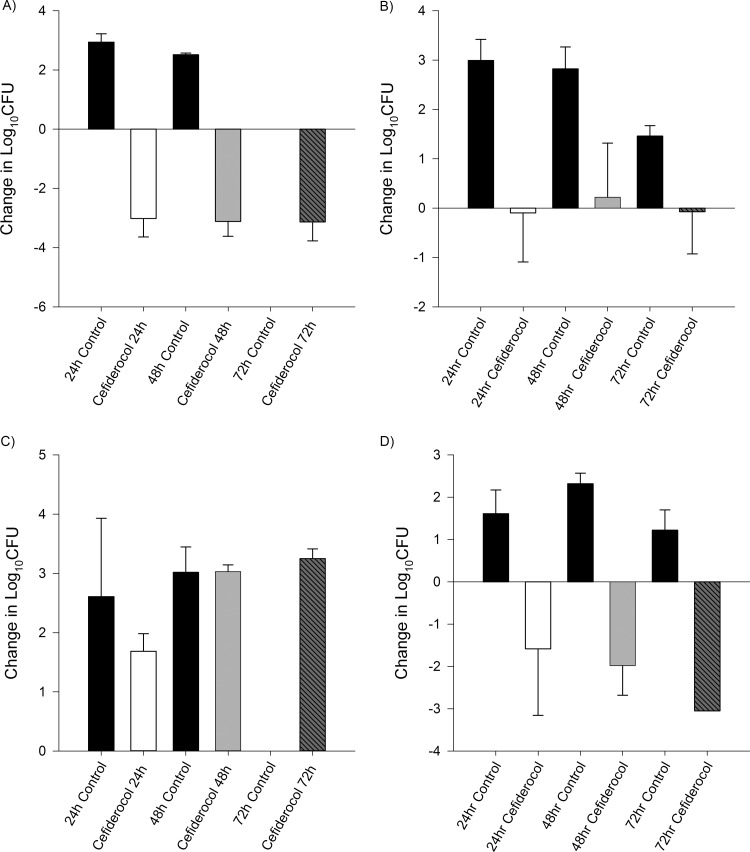
Efficacy of human-simulated exposures of cefiderocol compared with same-period untreated controls at 24, 48, and 72 h among A. baumannii isolates. Isolate cefiderocol preexposure MICs (μg/ml): A. baumannii 135, 0.5 (A); A. baumannii 152, 1 (B); A. baumannii 84, 16 (C); A. baumannii 87, 4 (D). Absence of control data at any given interval indicates zero survival for that isolate.

**FIG 2:**
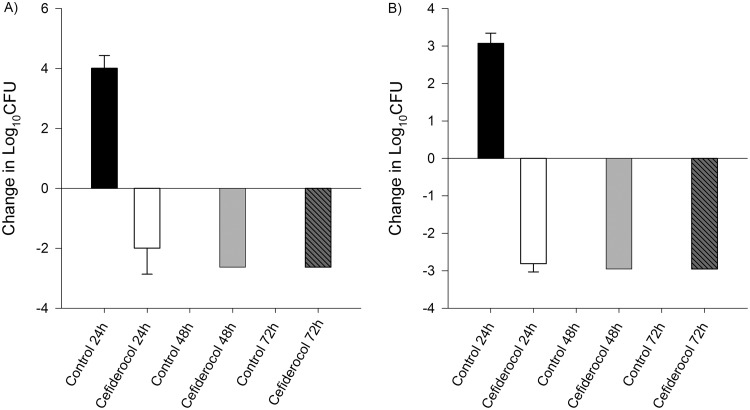
Efficacy of human-simulated exposures of cefiderocol compared with same-period untreated controls at 24, 48, and 72 h among P. aeruginosa isolates. Isolate cefiderocol preexposure MICs (μg/ml): P. aeruginosa 1574, 2 (A); P. aeruginosa 1595, 4 (B). Absence of control data at any given interval indicates zero survival for that isolate.

**FIG 3:**
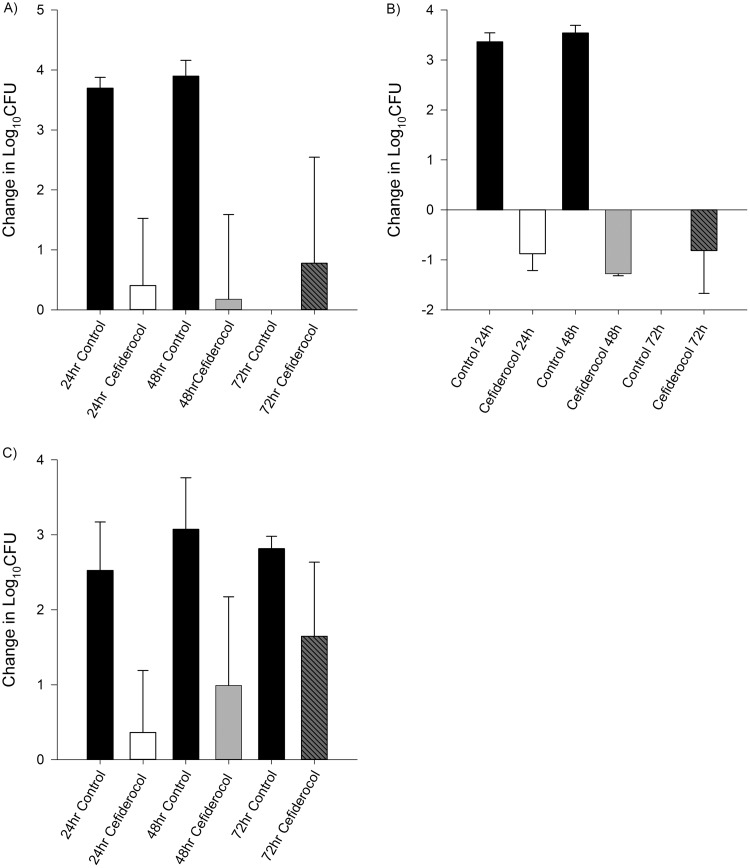
Efficacy of human-simulated exposures of cefiderocol compared with same-period untreated controls at 24, 48, and 72 h among Enterobacteriaceae. Isolate cefiderocol preexposure MICs (μg/ml): K. pneumoniae 531, 4 (A); K. pneumoniae 519, 4 (B); E. coli 462, 1 (C). Absence of control data at any given interval indicates zero survival for that isolate.

**FIG 4:**
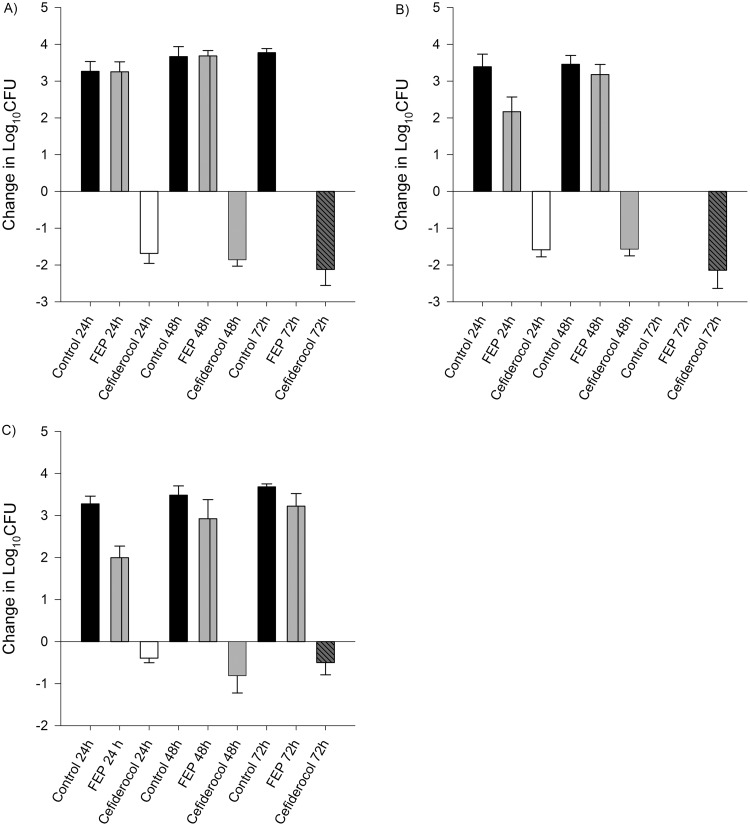
Efficacy of human-simulated exposures of cefiderocol and cefepime compared with same-period untreated controls at 24, 48, and 72 h among Enterobacteriaceae. Isolate cefiderocol preexposure MICs (μg/ml): E. coli 458, 1/512 (A); K. pneumoniae 539, 4/>512 (B); K. pneumoniae 543, 8/>512 (C). Absence of control data at any given interval indicates zero survival for that isolate.

Average initial bacterial burden at 0 h among the isolates was 6.08 ± 0.25 log_10_ CFU/thigh for A. baumannii, 5.49 ± 0.23 log_10_ CFU/thigh for P. aeruginosa, 5.91 ± 0.44 log_10_ CFU/thigh for E. coli, and 5.92 ± 0.24 log_10_ CFU/thigh for K. pneumoniae. In isolates displaying initial kill at 24 h after human-simulated exposures of cefiderocol, a sustained antimicrobial effect was observed over the subsequent 48-h dosing period (Fig. 1 to 4). Cefepime-resistant isolates (EC 458, KP 539, KP 543; MIC, ≥512 μg/ml) showed kill on cefiderocol therapy, while validation of the model was confirmed as comparator animals receiving human-simulated exposures of cefepime grew >3 log_10_ CFU at 72 h (Fig. 4).

Results from the retest MICs after cefiderocol exposure showed a 2-dilution change in only one treated animal (1/54 samples [1.8%]). The MIC in this single sample increased from 1 to 4 μg/ml for isolate E. coli 462 at 72 h, while two additional samples from similarly infected animals with E. coli 462 showed no change after 72 h of cefiderocol exposure.

As expected, isolates 40a, 41a, and 42b shared the same sequence type and horizontally transferred β-lactamases as isolate 32a, namely, ST410 (ST23 complex) and *bla*_CMY-42_, respectively (Table 2). Similarly, isolate 82a had the same ST and β-lactamase genes as 73a, i.e., ST258 and *bla*_SHV-12_ and *bla*_KPC-2_, respectively.

**TABLE 2:** Whole-genome sequencing for selected isolates from infected animals after 72 h of no treatment or cefiderocol exposure

Isolate	Sample	Regimen at 72 h	MLST	β-Lactamase	Lesion relative to control
E. coli 462	32a	Control	410	*bla*_EC_; *bla*_CMY-42_	Not applicable
E. coli 462	40a	Cefiderocol	410	*bla*_EC_; *bla*_CMY-42_	None identified
E. coli 462	41a	Cefiderocol	410	*bla*_EC_; *bla*_CMY-42_	ΔpcnB, S100A in EMU65688.1
E. coli 462	42b	Cefiderocol	410	*bla*_EC_; *bla*_CMY-42_	None identified
K. pneumoniae 531	73a	Control	258	*bla*_SHV-12_; *bla*_KPC-2_	Not applicable
K. pneumoniae 531	82a	Cefiderocol	258	*bla*_SHV-12_; *bla*_KPC-2_	None identified

The single sample, E. coli 462 (sample 41a [Table 2]), which displayed elevated MICs to cefiderocol compared to the unexposed control isolate (sample 32a), is the only isolate in which mutations were identified coding for changes in annotated protein sequences. Notably, a 4-base deletion of “CCAG” was identified that resulted in a frameshift at amino acid position 54 in the RNA poly(A) polymerase gene *pcnB*. The other mutation found in 41a was an SNP in a transposase 38 nucleotides from the edge of contig 59. While the mutation did code for an amino acid change (S100A), it is notable that transposases and other multiple copy genes will be prone to erroneous mapping in *de novo* assembly. No deduced amino acid sequence changes were identified in the other cefiderocol-exposed samples of E. coli 462 (40a or 42b relative to 32a), nor were any changes found in K. pneumoniae 531 isolates (sample 82a relative to 73a [Table 2]), leaving the predicted deletion of *pcnB* in EC 462 sample 41 as the sole lesion identified in any of the postexposure bacteria.

Examples of adaptive resistance following siderophore-conjugated monobactam exposure are well documented (4, 5). In such cases, despite supratherapeutic exposure of the siderophore antibiotic, bacterial growth similar to that in control animals was observed at 24 h. Moreover, resistant mutants rapidly evolved (4). In the current study, following human-simulated exposure of cefiderocol at 24 h, regrowth to the level of control was not observed. Furthermore, continued treatment at 48 and 72 h showed sustained kill in most isolates. For bacteria demonstrating cumulative regrowth (i.e., E. coli 462 and K. pneumoniae 531), the pattern of regrowth over the initial 24-h treatment period was inconsistent with the emergence of resistance observed with other siderophores (4, 5). Moreover, the phenomenon of adaptive resistance was not observed over the extended 72-h treatment period. The underlying mechanism behind this discordance between *in vitro* susceptibility and *in vivo* regrowth is not well understood. Documented cases of persister cells in the context of Gram-negative organisms against conventional antibiotic agents have been described in the literature (14).

Previous investigations into the mutations noted in our single isolate revealed that deletion of this gene led to a large regulatory switch, leading to changes in expression in multiple systems, including downregulation of the ferrichrome receptor operon fhuABCD, although these mutational changes have failed to demonstrate a link to cefiderocol resistance (15, 16).

Among a diverse group of Gram-negative isolates with a range of cefiderocol MICs, humanized exposures of the compound displayed sustained *in vivo* antibacterial effects over a 72-h period. Moreover, postexposure MIC studies revealed that the phenotypic profile for cefiderocol remained largely unchanged. Taken together, our current findings further illuminate the pharmacodynamic profile of this novel siderophore against resistant Gram-negative organisms.
